# Optimized aortic root segmentation during transcatheter aortic valve implantation

**DOI:** 10.3389/fcvm.2025.1602780

**Published:** 2025-11-13

**Authors:** Nikita V. Laptev, Olga M. Gerget, Julia K. Belova, Evgeny E. Vasilchenko, Mikhail A. Chernyavskiy, Viacheslav V. Danilov

**Affiliations:** 1 Siberian State Medical University, Tomsk, Russia; 2 Institute of Control Sciences of Russian Academy of Sciences, Moscow, Russia; 3 Almazov National Medical Research Center, Saint Petersburg, Russia; 4 Pompeu Fabra University, Barcelona, Spain

**Keywords:** automatic segmentation, aortic root, angiographic images, TAVI, convolutional neural networks

## Abstract

Transcatheter aortic valve implantation (TAVI) is a highly effective treatment for patients with severe aortic stenosis. Accurate valve positioning is critical for successful TAVI, and highly accurate real-time visualization—with minimal use of contrast—is especially important for patients with chronic kidney disease. Under fluoroscopic conditions, which often suffer from low contrast, high noise and artifacts, automatic segmentation of anatomical structures using convolutional neural networks (CNNs) can significantly improve the accuracy of valve positioning. This paper presents a comparative analysis of various CNN architectures for automatic aortic root segmentation on angiographic images, with the aim of optimizing the TAVI process. The experimental evaluation included models such as FPN, U-Net++, DeepLabV3+, LinkNet, MA-Net, and PSPNet, all trained and tested with optimally tuned hyperparameters. During training dynamics, DeepLabV3+ and U-Net++ showed stable convergence with median Dice scores around 0.88. However, when evaluated at the patient level, MA-Net and PSPNet outperformed all other models, achieving Dice coefficients of 0.942 and 0.936, and an average symmetric surface distance of 4.1 mm. The findings underscore the potential of incorporating automatic segmentation methods into decision-support systems for cardiac surgery—reducing contrast agent use, minimizing surgical risks, and improving valve positioning accuracy. Future work will focus on expanding the dataset, exploring additional architectures, and adapting the models for real-time application.

## 1 Introduction

TAVI represents a vital alternative to conventional surgical aortic valve replacement, especially for patients with symptomatic severe aortic stenosis who are at high risk for open-heart surgery. The increasing prevalence of TAVI has broadened its indications (1). However, complications—often stemming from a mismatch between prosthesis size and the fibrous aortic ring (2, 3) or from improper device deployment (4)—remain a significant concern. Many post-operative complications are closely related to the experience of the operating surgeon. In addition, patient motion (e.g., chest excursion during respiration and cardiac activity) further complicates device implantation (5, 6). In addition, the development of complications largely depends on the quality of intraoperative imaging required for accurate valve placement (2).

Accurate intraoperative imaging is crucial for precise valve placement; yet, conventional methods impose limitations due to increased radiation exposure and the need for repeated contrast injections, which elevate the risk of renal complications. Consequently, developing systems that reliably identify key anatomical landmarks while minimizing contrast agent use and radiation exposure is of paramount importance.

Recent advances in visual support systems allow the integration of preoperative three-dimensional computed tomography (CT) models with intraoperative X-ray images (7, 8). Nonetheless, challenges such as patient movement, deformation of anatomical structures by rigid instruments, and low image contrast complicate direct comparisons between preoperative and intraoperative images (9–13).

To address these challenges, our approach integrates several strategies: multi-scale CNN architectures (e.g., U-Net++, DeepLabV3+) enhance boundary detection under low-contrast and noisy conditions; extensive data augmentation (random shifts, rotations, noise addition, perspective distortions) improves robustness against artifacts and patient motion; and the inclusion of lightweight yet accurate models (e.g., LinkNet, MA-Net) ensures computational efficiency suitable for intraoperative use. These design choices directly respond to the difficulties inherent in real-time angiographic segmentation.

Unlike prior studies, our work focuses on the automatic segmentation of the aortic root directly from individual fluoroscopic images captured during TAVI. This approach provides a rapid and accurate method for segmenting the aortic root, thereby simplifying procedural navigation and increasing safety. By combining state-of-the-art deep learning techniques with adaptations tailored to intraoperative imaging conditions, our method opens new avenues for optimizing TAVI procedures and improving clinical outcomes.

This paper provides a comparative analysis of six deep neural network architectures—FPN, U-Net++, DeepLabV3+, LinkNet, MA-Net, and PSPNet—for automatic aortic root segmentation from intraoperative angiographic images. The primary objective is to identify the model that delivers high segmentation accuracy with minimal contrast media use while maintaining computational efficiency. We also discuss hyperparameter tuning strategies and model optimization for deployment under constrained computing resources.

## 2 Materials and methods

The development of our segmentation system for aortic root segmentation followed a two-stage process:

- **Stage 1: Data preparation**
  ○ Data labeling and creation of training and verification sets.
  ○ Each fluoroscopic image was annotated independently by two experienced vascular surgeons. All annotations were then reviewed by the Head of the Department of Vascular and Interventional Surgery. In cases of disagreement or particularly complex anatomy, the final segmentation was established by consensus in a joint meeting of the annotators. This multi-observer approach ensured a high-quality ground truth segmentation for training and evaluation.
- **Stage 2: Training and evaluation**
  ○ Selection of CNN architectures, loss functions, and evaluation metrics.
  ○ Systematic evaluation of qualitative and quantitative parameters from training and validation datasets.

### 2.1 Data collection

During endovascular surgeries, including TAVI, angiography via fluoroscopy serves as the reference method for dynamic intraoperative imaging. Data were collected from intraoperative angiographs obtained between 2018 and 2024 during implantation procedures in 80 patients with severe aortic valve stenosis (Supplementary Table S1). The resulting dataset comprises 2,854 images (1,000×1,000 pixels, 8-bit grayscale). For the five-fold patient-level cross-validation, approximately 86%–88% of patients were assigned to the training set and 12%–14% to the validation/test set in each fold. Because the number of frames per patient varied, the exact ratio of training vs. test images fluctuated slightly across folds.

As part of the TAVI procedures, a series of anonymized images were obtained, illustrating four main procedural stages: (i) overview angiography (Supplementary Figure S1A), (ii) positioning of the catheter and delivery system (Supplementary Figure S1B), (iii) initiation of retraction of the delivery system and valve exposure (Supplementary Figure S1C), and (iv) control angiography after valve implantation (Supplementary Figure S1D). These images provide a comprehensive representation of procedural steps, aiding in the accurate assessment of device placement and function.

The dataset includes representative images of key procedural stages, such as valve positioning, initiation of device retraction with valve exposure, and complete valve deployment. Notably, some images depict the implantation of the *ACURATE neo valve* (14), while others show the *CoreValve Evolut R* (15)—both self-expanding, supra-annular valves with porcine pericardium leaflets.

Since precise TAVI device positioning requires tracking anatomical landmarks relative to the native valve plane, the dataset was further annotated during contrast agent injections using the Supervisely web-based computer vision platform (16).

### 2.2 Model selection

In this study, we evaluated six CNNs for aortic root segmentation in angiography images: U-Net++ (17), LinkNet (18), FPN (19), PSPNet (20), DeepLabV3+ (21), and MA-Net (22). These models were selected based on their proven performance in analyzing complex biomedical images.

U-Net++ is an advanced version of the U-Net architecture tailored for medical image segmentation. It employs a deeply controlled encoder-decoder structure with nested dense transitions between the encoder and decoder, enabling the capture of fine details. Its effectiveness has been demonstrated in numerous studies, including the semantic segmentation of coronary vessel X-ray images (23).

In addition to U-Net++, we employed LinkNet and FPN. LinkNet is a lightweight network that uses skip connections to efficiently recombine fine details from the encoder to the decoder. FPN, characterized by its top-down architecture and lateral connections, creates a feature pyramid that enhances the segmentation process.

PSPNet and DeepLabV3+ were chosen for their ability to process features at multiple scales and improve contextual awareness-qualities essential for accurately segmenting complex intravascular images, where distinguishing foreground from background is challenging (24).

Finally, MA-Net, the most modern CNN in our selection, integrates attention mechanisms to focus on the most salient features of the image, thereby increasing segmentation accuracy. This model effectively exploits the strengths of conventional CNN architectures while optimizing feature extraction and presentation.

Although not included in our evaluation, we acknowledge the emergence of transformer-based segmentation models such as TransUNet, Swin-Unet, and SegFormer (25–27). These architectures leverage global self-attention and have demonstrated promising results on various segmentation tasks. However, we opted not to include them in this study due to several practical considerations. First, the self-attention mechanism in transformers has quadratic complexity (O($N^{2}$)) with respect to the number of image pixels, making it computationally prohibitive for our high-resolution angiographic images (approximately 1,000 × 1,000 pixels). Second, our dataset of 2,854 images (from 81 patients) is relatively small—transformer networks, which lack the strong spatial inductive biases of CNNs, generally require much larger training datasets to avoid overfitting. Third, in an intraoperative clinical setting like TAVI, the need for efficient, near-real-time inference favors using lightweight CNN architectures that can run quickly on available hardware. Moreover, the six CNN models we selected already achieve excellent segmentation accuracy in our experiments (Dice coefficient up to 0.88), indicating that our performance requirements can be met without the added complexity of transformers. For these reasons, we focused on CNN-based models in this comparative analysis. The exploration of transformer-based segmentation approaches is deferred to future work when larger datasets and greater computational resources become available.

### 2.3 Hyperparameter tuning strategy

For aortic root segmentation, we carefully configured six segmentation networks—U-Net++, LinkNet, FPN, PSPNet, DeepLabV3+, and MA-Net. Achieving the optimal training settings for these models required a rigorous hyperparameter tuning process, during which each model underwent over 200 configuration tests to ensure optimal performance.

Our tuning process aimed to maximize the segmentation score, specifically focusing on the Dice Similarity Coefficient (DSC). To this end, we employed the DSC loss function (Equation 1), defined as follows:

$$\text{Loss}=1-\frac{2\sum \left(y_{\text{true}}\times y_{\text{\textbackslash{},pred}}\right)+\epsilon }{\sum y_{\text{true}}+\sum y_{\text{\textbackslash{},pred}}+\epsilon }$$ (1)

where, $y_{\text{true}}$ and $y_{\text{\textbackslash{},pred}}$ denote the true and predicted label values, respectively, and ϵ is a small constant (set to $10^{-7}$) for numerical stability to prevent division by zero.

Recognizing that not all hyperparameters impact model performance equally (28), our focus was on optimizing the most critical parameters: the encoder architecture, input image size, optimizer selection, and learning rate. Parameters such as batch size, activation functions, optimizer parameters, and convolution kernel sizes were kept constant. Table 1 provides a detailed summary of the hyperparameters studied and their corresponding values during model tuning.

**Table 1:** Hyperparameters used in network optimization.

Hyperparameter	Value	Count
Architecture	Unet++, LinkNet, FPN, PSPNet, DeepLabV3+, MA-Net	6
Encoder	EfficientNet-B0, EfficientNet-B4, EfficientNet-B0, SE-ResNeXt50, ResNet-50, ResNet-101, SE-ResNeXt101, RegNetX-120	8
Input size	512×512, 624×624, 896×896	3
Optimizer	Adam, Radam, RMSprop	3
Learning rate	$10^{-3}$, $10^{-4}$, $10^{-5}$	3

Hyperparameter optimization was conducted using a combination of Bayesian optimization and an early termination strategy. Instead of traditional random or grid search methods, we utilized the Optuna (29) library with the Tree-structured Parzen Estimator algorithm, which builds a probabilistic model of the hyperparameters to identify the most promising combinations for further testing. Additionally, early termination of unpromising configurations was implemented using Hyperband Pruner (30). This combination of methods, corresponding to the BOHB approach (31), provided enhanced computational efficiency and reliability compared to conventional hyperparameter optimization techniques.

### 2.4 Model training strategy

After determining the optimal hyperparameters, we trained and tested our models on the entire dataset. Due to the limited number of subjects (80 patients), we employed a 5-fold cross-validation approach. In each fold, data from 65 patients were used for training and 15 patients for testing (Supplementary Table S2). This partitioning scheme ensured that the subject groups in each subset remained mutually exclusive, thereby preventing any data leakage between training and testing sets.

During the setup and training stages, a series of augmentation transformations was applied using the *Albumentations* library (32). These transformations not only expanded the dataset but also served as a regularization method to mitigate overfitting. The augmentation workflow included:

- **Horizontal flip** with a 50% probability.
- **Shift, scale, and rotate** with a 50% probability: random shifts, scaling, and rotations within specified limits (shift limit = 0.0625, zoom limit = 0.1, rotation limit = 15).
- **Conditional filling**: padding images to ensure a consistent size for processing.
- **Gaussian noise** with a 20% probability: adding random noise with a variance range of 3–10.
- **Perspective distortion** with a 50% probability: applying random perspective transformations with a scale of 0.05–0.1.
- **Random brightness and contrast adjustment** with a 90% probability: adjusting brightness and contrast within limits (brightness limit = 0.2, contrast limit = 0.2).
- **Hue, saturation, and value adjustment** with a 90% probability: shifting hue, saturation, and value within specified limits (hue shift limit = 20, saturation shift limit = 30, value shift limit = 20).

Since the models vary in complexity, they require different amounts of GPU memory when using a fixed batch size. To ensure fair learning conditions, we adjusted the batch size so that each model utilized approximately 70%–90% of the available GPU memory.

All training, tuning, and testing were conducted on server hardware comprising a 40-core Intel(R) Xeon(R) Gold 5218R CPU @ 2.10 GHz, 512 GB of RAM, and an Nvidia A6000 GPU with 48 GB of video memory. The models were developed using PyTorch v2.1 and Python v3.11.

## 3 Results

### 3.1 Tuning hyperparameters

Each model underwent a rigorous hyperparameter tuning process as described in the Section 2.3, with over 200 configurations tested per model. The results obtained at the tuning stage are summarized below and detailed in Table 2:

**Table 2:** Optimal hyperparameters for the studied networks.

Architecture	Encoder	Input size	Optimizer	LR	Parameters, M	FLOPs, G	Config checked
U-Net++	EfficientNet-B4	896	RMSprop	0.0001 × 10^−4^	72.4	502.1	216
LinkNet	SE-ResNeXt101	512	RMSprop	0.001 × 10^−3^	17.9	53.2	215
FPN	SE-ResNeXt101	512	RMSprop	0.001 × 10^−3^	19.4	99.2	216
PSPNet	SE-ResNeXt101	512	Adam	0.001 × 10^−3^	47.7	24.2	216
DeepLabV3+	SE-ResNeXt101	512	RMSprop	0.0001 × 10^−4^	18.6	113.6	189
MA-Net	EfficientNet-B4	896	RMSprop	0.001 × 10^−3^	25.6	39.1	216

- **Encoder:** EfficientNet-B4 and SE-ResNeXt101 were the most commonly used encoders across the architectures. Specifically, U-Net++ and MA-Net employed EfficientNet-B4, while LinkNet, FPN, PSPNet, and DeepLabV3+ were based on SE-ResNeXt101.
- **Input data size:** Input dimensions were adapted for each model, ranging from 512×512 to 896×896 pixels. This variation reflects a trade-off between computational efficiency and the level of detail required for accurate segmentation.
- **Optimizer and learning rate:** Optimizer and Learning Rate: RMSprop was primarily used as the optimizer, with the exception of PSPNet, which employed Adam.
- **Learning rate:** The optimal learning rate depended on the model architecture, parameter count, and computational complexity. For more complex, resource-intensive models (e.g., U-Net++, DeepLabV3+), a lower learning rate (0.0001) was preferable to maintain training stability. For models with fewer parameters and moderate complexity (e.g., FPN, LinkNet, PSPNet, MA-Net), a learning rate of 0.001 allowed for faster training without degrading performance.
- **Accuracy:** Model performance was evaluated using the DSC on the validation subset, which measures the overlap between the model prediction and the true segmentation. DSC scores ranged from 0.906 (PSPNet) to 0.916 (FPN), indicating that FPN achieved the highest segmentation accuracy during the tuning phase.
- **Complexity:** The number of parameters and the floating-point operations per second (FLOPs) indicate each model’s computational requirements. FPN (19.35 million parameters, 99.2 G FLOPs), DeepLabV3+ (18.62 million, 113.52 G FLOPs), and LinkNet (17.86 million, 53.18 G FLOPs) have a relatively similar (and generally smaller) parameter count, though FPN and DeepLabV3+ require higher FLOPs compared to LinkNet due to their architectural features. U-Net++ exhibited the highest complexity, with 72.38 million parameters and 502.097 billion FLOPs, making it the most computationally intensive. PSPNet (47.69 million parameters, 24.19 G FLOPs) and MA-Net (25.63 million parameters, 39.06 G FLOPs) showed average resource consumption.
- **Stability and training time:** During hyperparameter tuning, DeepLabV3+ experienced 27 crashes, while LinkNet encountered only one. The remaining models completed all configurations without failures. Tuning times ranged from 177 h (LinkNet) to 499 h (DeepLabV3+), reflecting differences in model complexity, input size, and the number of configurations tested.

The hyperparameter tuning results indicate that FPN achieved the highest DSC scores, suggesting that it is the most suitable model for aortic root segmentation on the tuning verification subset. DeepLabV3+ followed closely, with DSC scores of 0.916 and 0.915, respectively, while U-Net++ and MA-Net exhibited comparable results at 0.913, albeit with a slightly higher computational load. Meanwhile, LinkNet and PSPNet provide a favorable accuracy-to-complexity ratio, making them particularly viable in scenarios with limited computing power or stricter processing time requirements.

### 3.2 Model training

The study conducted a comprehensive assessment of the performance and convergence characteristics of six deep learning models: U-Net++, LinkNet, FPN, PSPNet, DeepLabV3+ and MA-Net. The models were trained over 35 epochs with an analysis of the dynamics of the loss function and the DSC coefficient (Supplementary Figure S2). The research results revealed a consistent pattern in all models, demonstrating a gradual decrease in losses and a corresponding increase in the DSC coefficient throughout the learning process. These trends indicate the ability of models to learn and improve their segmentation capabilities as they learn.

Convergence was determined by the stabilization of both metrics (Supplementary Table S3). DeepLabV3+ demonstrated consistent loss reduction and DSC improvement, reaching convergence between epochs 10 and 15. MA-Net and U-Net++ also converged rapidly, though with minor fluctuations that suggest more complex optimization dynamics. In contrast, LinkNet converged at a slower pace, ultimately achieving a DSC close to 0.877, while PSPNet showed the slowest convergence with a lower median DSC of 0.854. FPN exhibited the least stable behavior—likely due to its architectural design or hyperparameter configuration.

A detailed performance analysis using DSC metrics revealed notable differences in model stability (Supplementary Figure S3). The lower bounds, defined by the 1.5⋅IQR whiskers, ranged from 0.754 for PSPNet to 0.817 for DeepLabV3+, with PSPNet displaying the widest spread (0.754–0.901) and the lowest central tendency. DeepLabV3+ and U-Net++ showed more consistent behavior, with DSC values spanning from 0.817 to 0.913 and 0.826 to 0.913, respectively, indicating stable optimization trajectories. Median DSC scores clustered closely for the stronger models: U-Net++ (0.882) and DeepLabV3+ (0.881) achieved the highest central performance, followed by MA-Net (0.878) and LinkNet (0.877). Although FPN reached a similar maximum (0.913), its wider interval (0.794–0.913) reflects reduced stability compared to these models. PSPNet, with the lowest median DSC (0.854) and the broadest range, demonstrated the least reliable segmentation outcomes. Maximum DSC values, defined by the upper whisker, ranged from 0.901 (PSPNet) to 0.913 (U-Net++, FPN, DeepLabV3+, MA-Net), with LinkNet peaking at 0.909.

In summary, DeepLabV3+, U-Net++, and MA-Net emerged as the most balanced models in terms of accuracy and stability, while PSPNet and FPN may require further optimization to reduce variability. However, epoch-wise training analysis does not directly reflect patient-level robustness. To address this, we performed a separate evaluation across patients at the best epoch for each fold.

### 3.3 Patient-level evaluation

In addition to epoch-wise training dynamics, we performed a patient-level evaluation at the best-performing epoch for each fold (Table 3). This analysis provides a clinically oriented estimate of segmentation robustness. Median DSC values across patients ranged from 0.926 (U-Net++) to 0.942 (MA-Net), while ASSD values spanned 4.05–4.89 mm. Reported ASSD values are given in millimeters, using a fixed PixelSpacing of 0.390625 mm/pixel derived from the DICOM headers of the fluoroscopy system, which remained constant across all cases.

**Table 3:** Patient-level evaluation results with 95% bootstrap confidence intervals.

Model	DSC median	DSC 95% CI	ASSD median (mm)	ASSD 95% CI (mm)
DeepLabV3+	0.929	[0.915, 0.938]	4.619	[4.034, 5.578]
FPN	0.933	[0.924, 0.941]	4.371	[3.985, 5.469]
LinkNet	0.934	[0.920, 0.939]	4.567	[3.848, 5.420]
MA-Net	0.942	[0.934, 0.951]	4.067	[3.384, 4.387]
PSPNet	0.936	[0.929, 0.943]	4.051	[3.742, 4.910]
U-Net++	0.926	[0.917, 0.939]	4.894	[4.961, 5.511]

**Figure 1:**
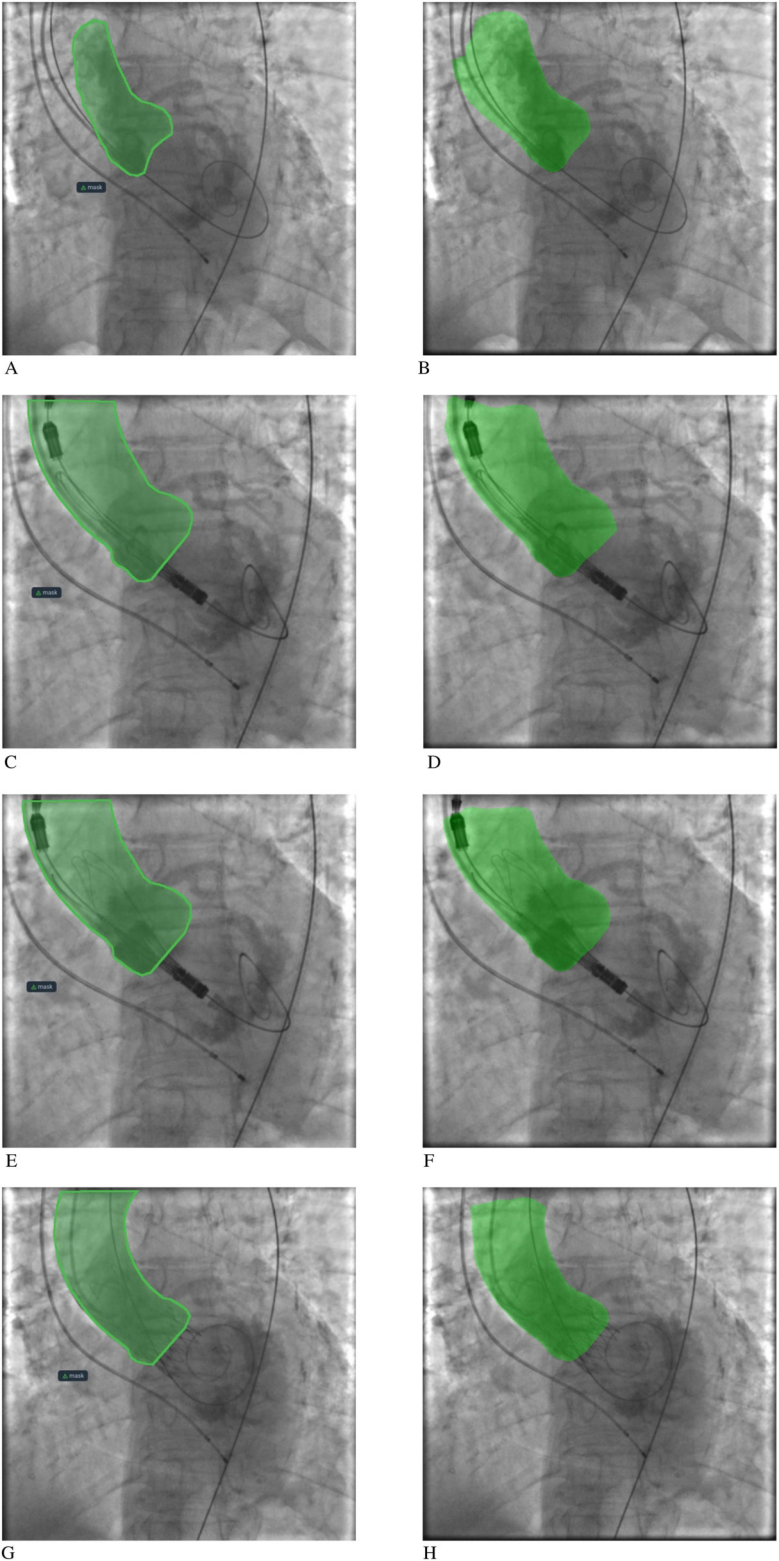
Aortic root segmentation results: **(A, C, E, G)** segmentation mask labeling by an interventional cardiologist; **(B, D, F, H)** segmentation mask labeling by the MA-Net model.

MA-Net (EfficientNet-B4) achieved the highest median DSC (0.942, 95% CI: 0.934–0.951) and one of the lowest ASSD scores (4.07 mm, 95% CI: 3.384–4.387). PSPNet (SE-ResNeXt101) produced a nearly comparable DSC (0.936, 95% CI: 0.93–0.94) and the lowest ASSD overall (4.05 mm, 95% CI: 3.742–4.910). LinkNet, FPN, and DeepLabV3+ achieved intermediate results (median DSC ≈ 0.93, ASSD 4.3–4.6 mm). U-Net++ ranked lowest with a median DSC of 0.926 (95% CI: 0.917–0.939) and the highest boundary error (ASSD 4.89 mm, 95% CI: 3.962–5.511). To compare model performance, we performed paired Wilcoxon signed-rank tests on the per-patient Dice scores with Holm correction for multiple comparisons. This analysis confirmed statistically significant differences between several model pairs, most notably between MA-Net and U-Net++, MA-Net and LinkNet, and PSPNet and U-Net++ (Supplementary Table S4). These results reinforce the superiority of MA-Net and PSPNet, not only during training dynamics but also when evaluated per patient, underscoring their clinical robustness. Figure 1 illustrates representative segmentation overlays produced by the MA-Net model, highlighting its promising performance.

## 4 Discussion

### 4.1 Segmentation performance and robustness

This study systematically benchmarks six modern CNN architectures for aortic root segmentation on intraoperative angiographic frames acquired during TAVI. In a low-contrast, artifact-prone setting, U-Net++ and DeepLabV3+ achieved the most favorable balance of accuracy and stability (median DSC ≈0.88), with MA-Net and LinkNet offering competitive performance at lower computational cost. These findings align with broader medical-imaging evidence that (i) densely connected U-Net variants better preserve small structures through multi-scale skip paths and deep supervision, and (ii) atrous-convolution backbones with pyramid pooling (as in DeepLabV3/+) improve context aggregation under limited contrast and variable object scales (33–35). While PSPNet and FPN reached high best-case scores during tuning, their wider DSC dispersion in cross-validation suggests sensitivity to hyperparameters and image quality fluctuations, a known challenge in fluoroscopic segmentation where noise, motion and overlapping devices degrade local gradients. At the patient level, MA-Net and PSPNet emerged as the most robust models, combining high overlap (Dice ≥ 0.936) with low boundary error (ASSD ≤ 4.1 mm). LinkNet, FPN, and DeepLabV3+ performed moderately, with Dice around 0.93 and ASSD between 4.3 and 4.6 mm. U-Net++ lagged behind, showing the lowest Dice (0.926) and highest ASSD (4.89 mm). In addition, statistical testing using paired Wilcoxon signed-rank tests with Holm correction demonstrated that differences between several models were statistically significant, reinforcing the robustness of MA-Net and PSPNet compared to others (Supplementary Table S4). Importantly, the patient-level evaluation highlighted that overlap-based and boundary-based metrics are not always aligned: while MA-Net maximized Dice, PSPNet minimized ASSD, pointing to complementary strengths in overlap accuracy vs. boundary precision. Together, these findings suggest that MA-Net and PSPNet are the most balanced and clinically reliable among the tested models, whereas U-Net++—despite strong performance during training-proved less consistent at the patient level.

### 4.2 Positioning within existing solutions

Most automation in TAVI image guidance has focused on pre-procedural CT: fully automatic 3D aortic-root (AR) segmentation, landmark detection (annulus, STJ), and measurement extraction can now reach Dice ≈ 0.90–0.93 and millimetric agreement to expert annotations, enabling accurate sizing and prediction of optimal C-arm angulation for implantation (36–38). Parallel clinical lines of work register these CT models to live fluoroscopy (CT-XR fusion) to overlay the annular plane and coronary ostia and to guide device trajectories; feasibility and workflow utility have been repeatedly demonstrated, including improved projection selection and targeted catheterization (and, in related structural cases, PVL closure guidance) (39, 40).

By contrast, purely fluoroscopy-based automation remains less explored. Prior angiographic deep-learning has concentrated on prosthesis or coronary vessel segmentation (often with DeepLabV3+-type decoders or custom pre-processing), rather than segmenting native aortic-root anatomy during valve deployment (24, 41, 42). Our results therefore complement CT-centric pipelines and fusion systems: they show that, even without CT, single-frame fluoroscopic segmentation of the aortic root is technically feasible at clinically meaningful overlap, and can be computed fast enough for intraoperative decision support when lightweight backbones are chosen.

### 4.3 Clinical relevance and potential impact

From a clinical perspective, accurate delineation of the aortic root on live angiography has three immediate implications:

1. **Projection and deployment control.** When the annular plane and sinuses are well segmented, operators can cross-check depth and coaxiality against the prosthesis in real time, particularly during rapid pacing or partial release. This complements CT-predicted C-arm angles and reduces reliance on repeated contrast runs to “re-find” the annulus in challenging anatomies (e.g., heavy calcification, horizontal aortas) (43).
2. **Complication mitigation.** Better intraoperative landmarking is mechanistically linked to less malpositioning-which in turn is a major driver of paravalvular leak, conduction disturbances and reintervention. While our study did not test clinical outcomes, CT-fluoro fusion literature already shows that improved landmark visualization facilitates device manipulation; by analogy, robust fluoro-native segmentation could offer similar intraoperative guardrails without the prerequisites of CT registration (39, 40).
3. **Contrast stewardship.** Repeated contrast injections for annulus re-identification contribute to Acute Kidney Injury risk (AKI), which is associated with worse short-term outcomes after TAVI. Multiple studies emphasize the importance of minimizing contrast volume and/or scaling it to renal function (e.g., contrast-to-eGFR ratios) to reduce AKI and mortality risk; tools that stabilize visualization at lower contrast loads are therefore clinically attractive (44–47).

### 4.4 Architectural trade-offs

Across architectures, two patterns emerged. First, multi-scale, dilation-based decoders (DeepLabV3+) and densely nested U-Net++ variants were consistently resilient to low SNR and background clutter-mirroring their documented strengths in other angiographic tasks that require long-range context with local boundary fidelity. Second, efficiency matters: LinkNet and MA-Net delivered respectable median DSC with markedly fewer FLOPs/parameters, which is relevant for real-time intraoperative deployment on commodity GPUs. These observations are congruent with the broader literature where tailored Deeplab/U-Net derivatives, sometimes preceded by contrast-normalization and denoising subnets, top benchmarks on X-ray angiography datasets.

### 4.5 Beyond CNNs: transformer and hybrid designs

Emerging transformer-based and hybrid models (e.g., Swin-DeepLab, TransDeepLab) may further enhance robustness to long-range dependencies and out-of-distribution artifact patterns. Early medical imaging studies suggest superiority over pure CNNs in heterogeneous datasets. Given dataset size and intraoperative latency constraints, CNNs were prioritized here; however, future work should evaluate compact transformer-CNN hybrids for potential deployment.

### 4.6 Validation strategy and next steps

Beyond cross-validated DSC, three axes of validation are critical:

- **Generalization and reproducibility.** External, multi-center testing across vendors and acquisition protocols, with reader-study assessment of anatomical plausibility (annular plane, coronary ostia proximity) and inter-observer agreement.
- **Task-linked endpoints.** Prospective studies that randomize or compare standard care vs. “segmentation-assisted” guidance should track projection changes, number of contrast runs, contrast-to-eGFR ratio, pacing time, device depth variance, and early outcomes (PVL grade, PPM implantation, 30-day AKI). Such endpoints have precedent in CT-fluoro fusion and AKI literature and can ground the technical metric in clinical effect size (40, 45).
- **Workflow integration.** Latency profiling and fail-safe design (confidence estimates with automatic fallback to manual workflow) are essential for OR adoption. In parallel, combining our fluoro-native segmentation with optional pre-procedural CT (when available) could offer a hybrid path: our mask stabilizes the annulus in noisy frames, while CT supplies pre-computed angles and 3D context (38).

### 4.7 Limitations

This study is limited by its single-center dataset and evaluation restricted to contrast-enhanced fluoroscopy frames. Non-contrast frames, extreme motion, and heavy device overlap remain challenging. Transformer hybrids were not benchmarked, and real-time performance was not tested under continuous cine acquisition. Most importantly, prospective outcome studies are required to establish clinical benefits beyond segmentation accuracy. The obtained results remain preliminary and should be considered as hypotheses awaiting confirmation in future multicenter studies based on the results.

## 5 Conclusion

This study demonstrates that U-Net++ and DeepLabV3+ achieve accurate, reliable aortic root segmentation during training, with stable convergence and consistent DSC performance. However, when evaluated on a patient-level basis, MA-Net and PSPNet outperformed all other models, combining the highest Dice values with the lowest ASSD errors. These results emphasize that patient-level evaluation provides a stricter and more clinically relevant measure of segmentation reliability.

By enabling reliable visualization under low-contrast and noisy imaging conditions, our approach aligns with clinical needs to minimize contrast exposure, especially important given the well-recognized association between contrast volume and post-TAVI renal injury. Our publicly released dataset, models, and code establish a reproducible foundation for fluoroscopy-based decision-support in TAVI.

Next steps include multicenter clinical validation, integration into real-time operating-room workflows, and quantitative assessment of procedural benefits, such as reduced contrast use, shorter procedural times, improved deployment accuracy, and better patient safety outcomes.

## Data Availability

The data supporting the key findings of this study are presented within the article/Supplementary material. All essential components of the study, including curated source code, data, and trained models, have been made publicly available: Source code: https://github.com/Nikita75699/segmentation_tavi. Dataset: https://doi.org/10.5281/zenodo.10838384. Models: https://doi.org/10.5281/zenodo.15106413.
