# Supplementary material for: Optimized aortic root segmentation during transcatheter aortic valve implantation
Source: Front Cardiovasc Med. 2025 Nov 13;12:1602780. doi: 10.3389/fcvm.2025.1602780 (PMC12657348; doi:10.3389/fcvm.2025.1602780)
Supplement: Supplementary file 1 [file Datasheet1.pdf]

Supplementary Material

Correspondence*:
Nikita V. Laptev, Olga M. Gerget, Viacheslav V. Danilov
nikitalaptev77@gmail.com, olgagerget@mail.ru, viacheslav.v.danilov@gmail.com

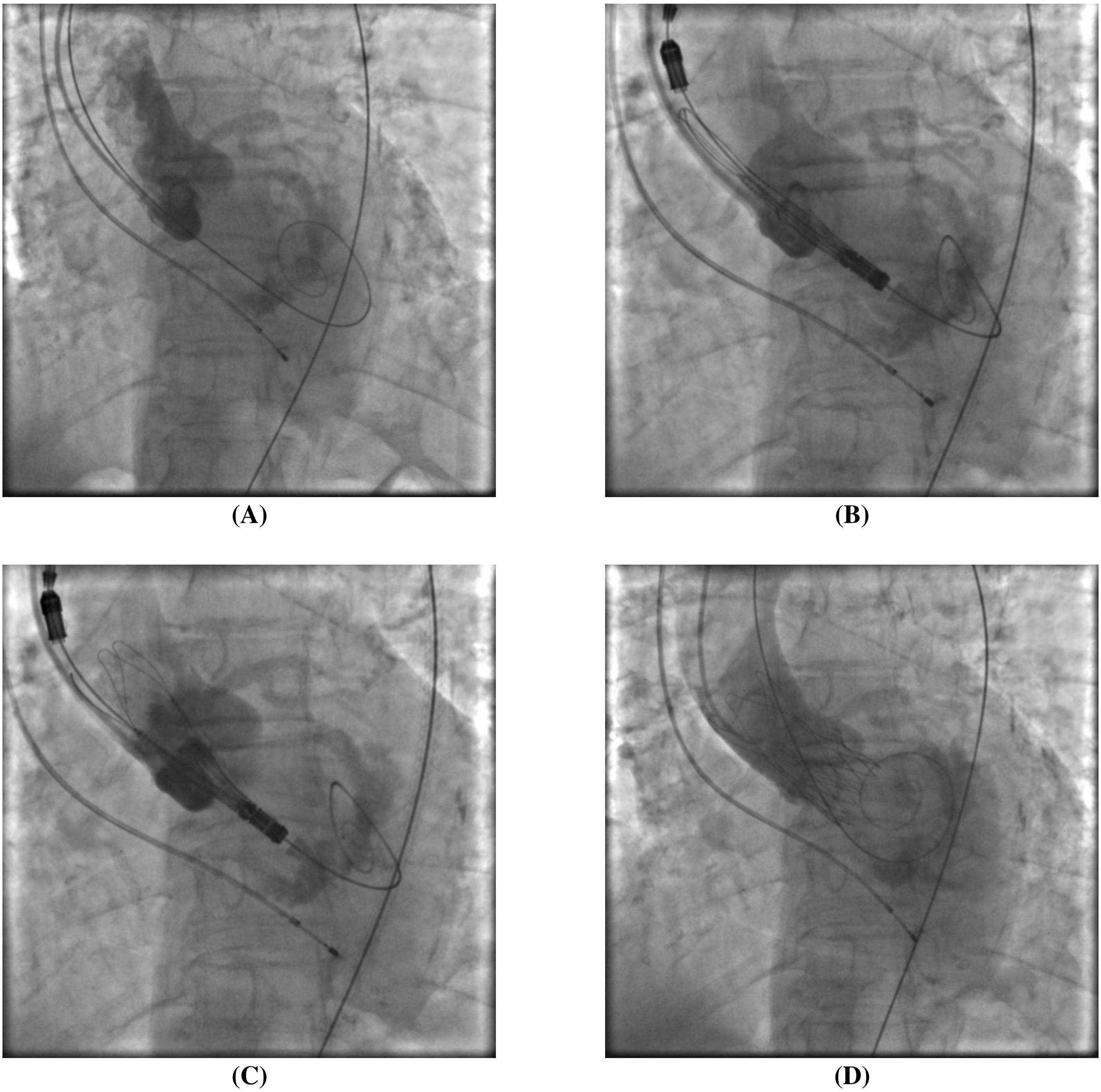

Figure 1. Data for labeling intraoperative aortography images during the TAVI procedure. **(A)** Overview angiography; **(B)** Positioning of the aortic valve; **(C)** Partial valve opening from the delivery system; **(D)** Follow-up angiography after valve implantation.

Table 1. Patient demographics and procedural details.

Parameter	Value
Total number of procedures	80
Mean age (mean ± SD), years	76.3 ± 5.8
Male patients, n (%)	28 (35%)
Female patients, n (%)	52 (64%)
Transfemoral access, n (%)	80 (100%)

Table 2. The distribution of patients by subsets.

Fold	Subset	Total
1	Train	2455
	Test	399
2	Train	2477
	Test	377
3	Train	2514
	Test	340
4	Train	2476
	Test	378
5	Train	2471
	Test	383

Table 3. Dynamics of convergence of the studied models.

Model	Convergence, epoch	Loss convergence	DSC convergence	DSC median
U-Net++	20-25	0.126	0.874	0.881
LinkNet	25-30	0.136	0.865	0.871
FPN	25-30	0.132	0.868	0.881
PSPNet	25-30	0.176	0.825	0.878
DeepLabV3+	15-23	0.123	0.887	0.877
MA-Net	20-25	0.127	0.873	0.854

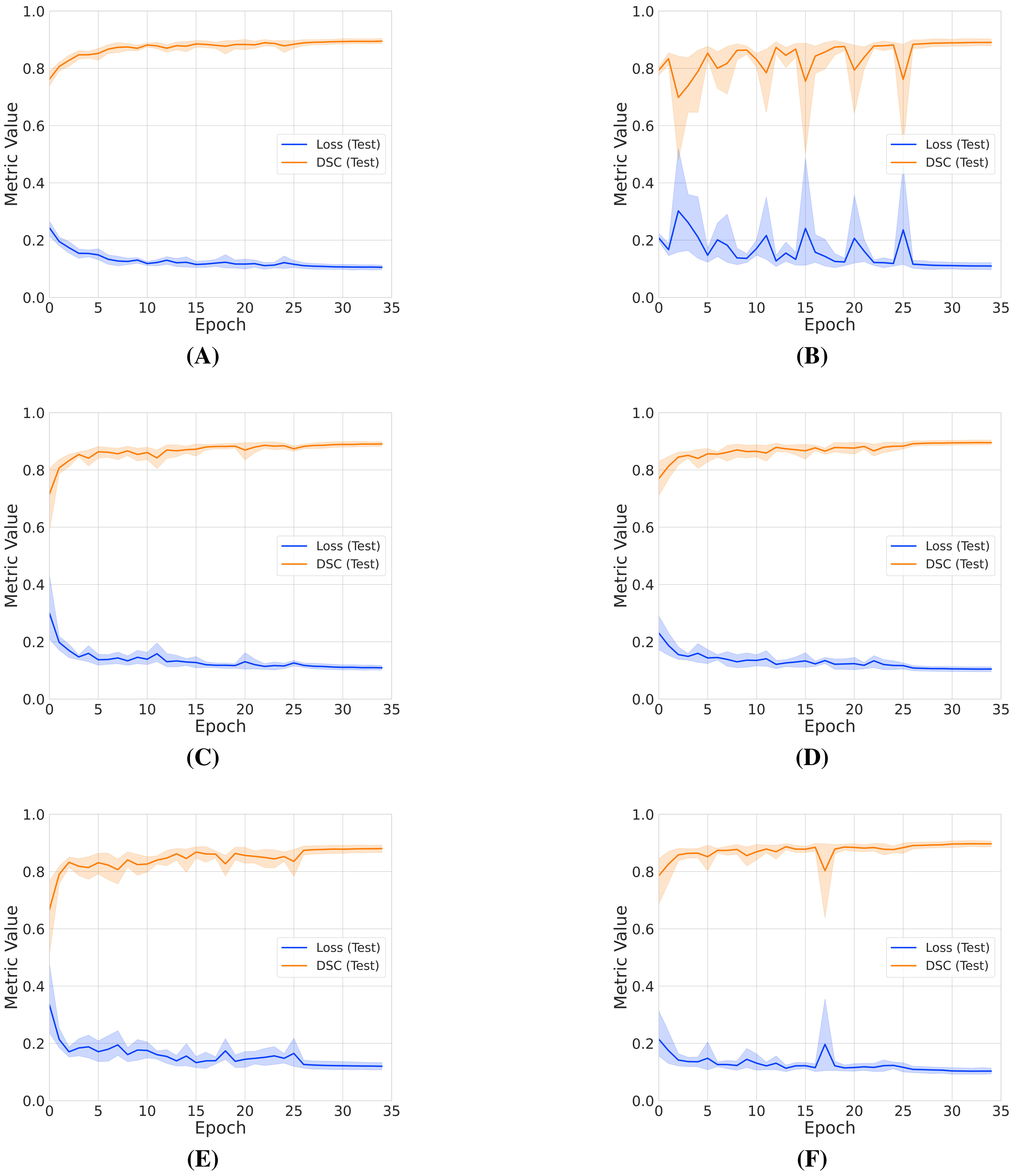

Figure 2. Comparative analysis of DSC losses and evolution during the training and testing phases with 5-fold cross-validation with 95% confidence interval. The models being compared include: **(A)** DeepLabV3+, **(B)** FPN, **(C)** LinkNet, **(D)** MA-Net, **(E)** PSPNet, **(F)** U-Net++.

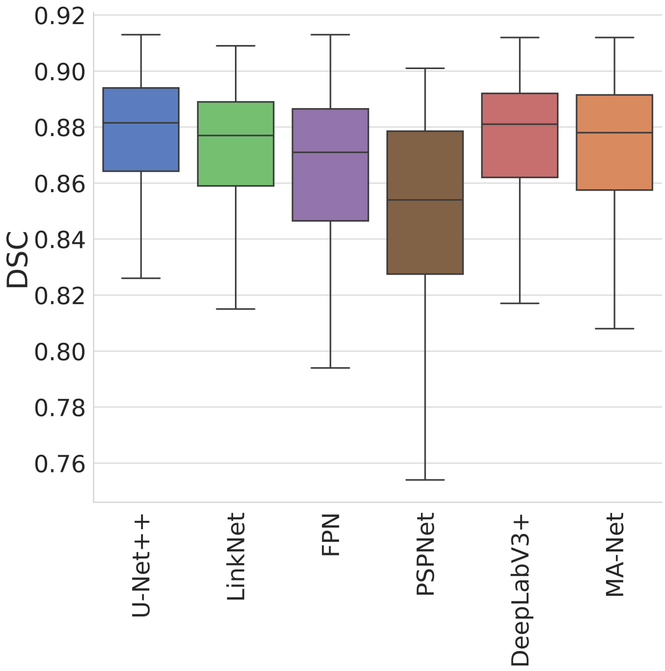

Figure 3. Average feature-wise performance across various models.

Table 4. Paired Wilcoxon signed-rank tests for DSC with Holm correction (per-patient CV).

**Model 1**	**Model 2**	**Raw** p	**Holm** p	**Significant** ($\alpha = 0.05$)
DeepLabV3+	FPN	3.00×10^{-2}	2.99×10^{-1}	No
DeepLabV3+	LinkNet	9.40×10^{-1}	9.40×10^{-1}	No
DeepLabV3+	MA-Net	2.14×10^{-8}	3.21×10^{-7}	Yes
DeepLabV3+	PSPNet	2.53×10^{-5}	2.78×10^{-4}	Yes
DeepLabV3+	U-Net++	1.99×10^{-1}	7.96×10^{-1}	No
FPN	LinkNet	3.71×10^{-1}	7.42×10^{-1}	No
FPN	MA-Net	1.04×10^{-6}	1.25×10^{-5}	Yes
FPN	PSPNet	2.00×10^{-3}	1.40×10^{-2}	Yes
FPN	U-Net++	9.04×10^{-1}	9.97×10^{-1}	No
LinkNet	MA-Net	1.44×10^{-8}	2.01×10^{-7}	Yes
LinkNet	PSPNet	2.98×10^{-6}	2.98×10^{-5}	Yes
LinkNet	U-Net++	2.94×10^{-1}	9.97×10^{-1}	No
MA-Net	PSPNet	2.59×10^{-4}	2.00×10^{-3}	Yes
MA-Net	U-Net++	5.93×10^{-6}	7.12×10^{-5}	Yes
PSPNet	U-Net++	1.00×10^{-3}	1.00×10^{-2}	Yes
